# Atualização no tratamento das lesões meniscais

**DOI:** 10.1055/s-0045-1814083

**Published:** 2025-11-21

**Authors:** Rafael Erthal de Paula, Bernardo Crespo Alves, Alan de Paula Mozella

**Affiliations:** 1 Centro de Cirurgia do Joelho, Instituto Nacional de Traumatologia e Ortopedia (INTO), Rio de Janeiro, RJ, Brasil

**Keywords:** menisco, artroscopia, traumatismos do joelho, joelho, procedimentos cirúrgicos operatórios, meniscus, arthroscopy, knee injuries, knee, surgical procedures operative

## Abstract

Ao longo das últimas décadas, a evolução do tratamento cirúrgico das lesões meniscais tem sido notável, especialmente à medida que um entendimento mais profundo das funções desempenhadas pelo menisco vem à tona. Nesse contexto, observa-se uma tendência a realizar abordagens cirúrgicas mais conservadoras, centradas na preservação da anatomia meniscal da forma mais próxima possível ao estado nativo. Isso se traduz em uma preferência por procedimentos de reparo meniscal, o que representa uma mudança significativa em relação às práticas anteriores. Essa transformação reflete o compromisso contínuo de otimizar os resultados clínicos e funcionais para os pacientes que enfrentam lesões meniscais.

## Abstract (en)

Throughout the last few decades, the evolution of the surgical treatment of meniscal injuries has been remarkable, especially since a deeper understanding of the meniscus functions has come to light. This evolution resulted in a trend towards more conservative surgical approaches, focusing on preserving the meniscal anatomy as close as possible to the native status. Such a trend has led to a preference for meniscal repair procedures, which represents a significant change from previous practices. This transformation reflects the ongoing commitment to optimize the clinical and functional outcomes for patients with meniscal injuries.

## Introdução

O menisco consiste em uma estrutura fibrocartilaginosa de significativa importância na manutenção da homeostase articular, e desempenha um papel fundamental em diversas funções; entre elas, destacam-se a distribuição de carga, a absorção de impacto, a estabilização da articulação, a propriocepção e a lubrificação articular. No entanto, essas funções vitais podem ser comprometidas de maneira substancial após a realização de uma meniscectomia radical. As lesões que acometem o menisco podem ser categorizadas em traumáticas e degenerativas, de modo que abrangem uma ampla gama de situações clínicas. A abordagem terapêutica dessas lesões varia de opções conservadoras até intervenções cirúrgicas. 1

## Classificação das Lesões

As lesões meniscais podem ser classificadas de acordo com sua morfologia, localização, orientação e extensão.

Quanto à localização e à extensão, as lesões podem ser dos cornos anterior e posterior, do corpo, e das raízes anterior e posterior. Essa classificação topográfica foi modificada por Smigielski et al., 2 que subdividiram as regiões segundo suas peculiaridades.

Quanto à orientação, as lesões podem ser longitudinais, quando se apresentam ao longo da circunferência do menisco, paralelas às fibras centrais, ou radiais, que são verticais, perpendiculares à circunferência, e cruzam as fibras centrais. A lesão longitudinal ainda pode ser horizontal (separa o menisco em uma porção superior e inferior), oblíqua ou vertical. As lesões verticais podem gerar um grande fragmento que se desloca para o intercôndilo, a chamada “lesão em alça de balde”. As lesões radiais podem ser retas ou curvas; estas últimas acarretam a formação de *flaps* . Lesões com apresentações anômalas, em múltiplas direções, são chamadas de complexas. 3

As lesões meniscais também podem ser classificadas pela ressonância magnética (RM) em graus I, II e III. A lesão de grau I é globular, e apresenta hipersinal intrameniscal que não se estende às superfícies articulares. Ela representa as alterações degenerativas iniciais do menisco. A lesão de grau II apresenta extensa área de hipersinal, sem plano de clivagem definido, e pode ou não se estender até as margens do menisco, mas não apresenta nítida comunicação com a cavidade articular; estas configuram alterações degenerativas mais avançadas. Já a lesão de grau III apresenta hipersinal bem definido, comunica a lesão às superfícies articulares, e denota uma lesão de origem traumática. 4

## Tratamento Conservador

O tratamento conservador é indicado em pacientes oligossintómaticos com lesões estáveis e como primeiro tratamento em lesões meniscais degenerativas. O achado de imagens compatíveis com lesão meniscal nos exames de imagem não é raro, mesmo em pacientes assintomáticos, e deve-se ter cuidado para não se superindicar o tratamento cirúrgico, 5 principalmente pela falta de evidências de que o resultado cirúrgico das meniscectomias parciais seja superior ao do tratamento conservador no longo prazo. Assim, a abordagem cirúrgica é mais frequentemente indicada diante de sintomas mecânicos e em lesões com padrão de instabilidade, como *flaps* ou alças de balde. 6 7 8

O tratamento conservador inclui o manejo com fisioterapia voltado para a melhora do quadro álgico, associado ao tratamento de déficits musculares e proprioceptivos, e em combinação com um programa de exercícios supervisionados, centrados no fortalecimento muscular dos quadris e joelhos. 9 10

## Tratamento Cirúrgico

### Meniscectomia Parcial

A meniscectomia parcial é uma cirurgia indicada em lesões em que o reparo com técnicas de sutura não é possível, ou em lesões com baixo potencial de cicatrização. Com o melhor entendimento da função meniscal, a abordagem deve ser restrita à área acometida, e deve-se procurar uma ressecção mínima, que permita a obtenção de bordas residuais firmes e regulares. Instrumentos manuais, como tesouras artroscópicas de diversas orientações ou instrumentais motorizados, como o microdebridador são utilizados para o debridamento das porções meniscais lesadas.

Geralmente, o pós-operatório é pouco exigente, com carga e mobilidade permitidas precocemente. Embora a meniscectomia parcial esteja associada com a melhora dos sintomas no curto prazo, gera mudanças biomecânicas no joelho e acarreta risco aumentado de degeneração articular quando comparada às técnicas que envolvem sutura, e pode estar associada a perda aguda de absorção de impacto e alteração da homeostase do osso subcondral, a ponto de evoluir para quadros de fratura por insuficiência ou osteonecrose pós-artroscopia.

## Meniscorrafia

### Sutura *Outside-in*

As técnicas de sutura *outside-in* possibilitam a execução de reparos que utilizam materiais simples e amplamente disponíveis, o que reduz os custos da técnica. São especialmente úteis em lesões localizadas nas regiões mais anteriores dos meniscos, e apresentam limitações para uso nas lesões mais posteriores. Podem ser executadas por meio da passagem de agulhas direcionadas para a área que se deseja reparar de fora para dentro, levando assim fios de sutura que podem ser resgatados para os portais artroscópicos, o que permite o transporte de um dos fios a ser utilizado para a sutura através da localização desejada. 11 12 Uma alternativa técnica envolve a utilização de uma agulha que leva um fio dobrado em forma de alça, que transportará outro fio a ser passado por uma segunda agulha introduzida com a mesma técnica; mediante a manipulação das agulhas, pode-se fazer a passagem entre os fios sem resgatá-los pelos portais. 13

Após a passagem do fio de sutura conforme desejado, um pequeno acesso pode ser realizado no local para que a sutura possa ser amarrada. Ao se realizar esta etapa, deve-se verificar a inexistência de estruturas anatômicas, tal como o nervo safeno, para evitar que sejam amarradas entre as pontas da sutura. Alternativamente, os fios podem ser recuperados pelos portais após a dissecção subcutânea com uma pinça artroscópica de tipo *grasper* , o que permite que o nó seja amarrado e direcionado com o uso de empurradores de nós artroscópicos. 14

### Sutura *Inside-out*

Técnica na qual agulhas para sutura meniscal são direcionadas através de cânulas de dentro da articulação para a região externa e recuperadas por acessos realizados nas regiões medial ou lateral, de modo a transportar os fios de sutura que serão usados na confecção dos pontos. A técnica tem um custo agregado devido à necessidade do uso das agulhas de sutura e dos fios cirúrgicos; entretanto, um mesmo *kit* com agulha pode ser utilizado para realizar diversos pontos, o que permite bastante versatilidade no direcionamento e no número de pontos. É considerada a técnica padrão-ouro. 11 15

A técnica tem como desvantagem a necessidade da realização de acessos cirúrgicos para proteger estruturas nobres que poderiam ser perfuradas ou inadvertidamente aprisionadas entre os nós. Na região medial, um acesso é recomendado para a proteção do nervo safeno e, na região lateral, da mesma forma, o acesso é recomendado, desta vez, com a finalidade de proteger o nervo fibular. A localização do acesso medial é planejada posteriormente ao ligamento colateral medial com cerca de 3 cm a 6 cm, sendo um terço do ligamento proximal à linha articular, e dois terços, distal, e deve ser realizada dissecção profunda no plano entre o gastrocnêmio medial e a borda posterior da tíbia. Instrumentais similares a uma colher podem ser utilizados para afastar e direcionar as agulhas que caminham em direção posterior durante a progressão a partir da articulação. 15

Na região lateral, o acesso indicado é realizado com cerca de 3 cm a 6 cm centrado na interlinha articular posteriormente ao ligamento colateral lateral, entre o bíceps femoral e o trato iliotibial. Uma dissecção dos planos profundos é realizada no intervalo entre o gastrocnêmio lateral e a cápsula articular, o que permite proteger as estruturas neurovasculares da região durante o direcionamento e o resgate das agulhas.

A técnica *inside-out* permite o reparo adequado de lesões do corpo e da região posterior meniscal, mas apresenta como desvantagens a necessidade da realização de acesso e de um auxiliar capacitado para executar o passo a passo da técnica, assim como um aumento do tempo cirúrgico quando comparada às técnicas *all-inside* .

### Sutura *All-inside*

Os dispositivos de sutura *all-inside* são materiais específicos para sutura meniscal que permitem a inserção de pontos através da articulação sem a necessidade de promover acessos para resgatar os fios de sutura. A primeira geração da técnica de sutura meniscal era realizada com a inserção de ganchos curvos para sutura através de portais artroscópicos posteriores e confecção de pontos pela técnica de amarria, com nós artroscópicos. 16

Com a evolução dos materiais disponíveis, uma segunda geração da técnica permitiu a utilização de dispositivos próprios que ancoravam os fios na cápsula através de um acesso pelos portais convencionais e exigiam, de modo similar à primeira geração, a execução dos nós pelo cirurgião com técnica artroscópica. 17

A terceira geração de dispositivos utilizava fios acoplados a dardos rígidos que se ancoravam na cápsula articular, o que permitia o tensionamento das suturas para a realização do reparo planejado. Esses dispositivos tinham a desvantagem de propiciar dano condral devido ao tamanho e à rigidez dos dardos. 18 19

Desenvolveu-se uma quarta geração de dispositivos, que superava as desvantagens dos dispositivos de terceira geração e melhorava os resultados da técnica. Trata-se de materiais específicos, com fios acoplados a materiais de baixo perfil utilizados para a ancoragem periférica das suturas e nós já armados, que permitem o tensionamento ao apertar a sutura conforme a necessidade. Esses dispositivos proporcionam resultados equivalentes aos conseguidos com as técnicas *inside-out* , com maior comodidade para o cirurgião e economia de tempo cirúrgico; entretanto, têm um custo aumentado, o que limita a sua utilização. 11 20

Recentemente, foram desenvolvidos novos dispositivos, que possibilitam a modelagem das hastes que lançam os sistemas de ancoragem acoplados aos fios, o que permite, assim, alcançar lesões localizadas em regiões de acesso mais difícil. Além desses dispositivos, materiais específicos para a realização de suturas *all-inside* com transfixação do tecido meniscal, sem a necessidade de ancoragem periférica capsular, foram criados, e possibilitam a execução de reparos circunferenciais que podem ser realizados em diversas configurações, de acordo com o planejamento cirúrgico. Esses instrumentais também facilitam a execução da reinserção meniscal transóssea com as técnicas de *pull-out* no tratamento das lesões das raízes meniscais.

## Situações Especiais

### Tratamento das Lesões Radiais

A lesão meniscal radial consiste em uma lesão vertical do menisco, perpendicular ao eixo circunferencial meniscal, que se inicia a partir da borda livre do menisco e corre para a sua porção periférica. Esta lesão pode ser parcial, quando não chega à junção meniscocapsular, ou completa, quando toda a estrutura do menisco está envolvida, e ele é separado em dois fragmentos (anterior e posterior à lesão). 21

O tratamento da lesão radial evoluiu com o melhor entendimento da função do menisco e os avanços técnicos em cirurgia. Nas primeiras descrições deste padrão de lesão, em 2004, Bin et al. 22 relataram que o reparo era praticamente impossível, devido à qualidade meniscal e ao baixo potencial de cicatrização. No entanto, bons resultados vêm sendo obtidos nas suturas dessas lesões, com melhora sintomática e restauração funcional. 23

Por muito tempo, a meniscectomia foi considerada o tratamento padrão para esse tipo de lesão. 24 Em lesões parciais, que acometem a porção central e avascular do menisco, o debridamento até a obtenção de uma borda estável e de contornos arredondados é um tratamento adequado, pois mantém o máximo de menisco nativo e reduz o risco de progressão periférica da lesão. Porém, devido às evidências de alterações na biomecânica da distribuição de carga e de pressão, mesmo diante de pequenas ressecções meniscais, e avanços nas técnicas de reparo meniscal, observa-se, mais recentemente, um aumento da tendência a realizar suturas nessas lesões. 25

O objetivo do reparo das lesões radiais é reestabelecer a capacidade de absorção do estresse axial, a transmissão de carga e evitar a extrusão meniscal. Os métodos de sutura das lesões meniscais apresentaram grande evolução, com diversos dispositivos para a realização das suturas *inside-out* , *outside-in* e *all-inside* . Diante de lesões meniscais complexas, como as radiais, o domínio das técnicas é importante, pois muitas vezes a combinação de técnicas é necessária para o tratamento adequado da lesão.

O padrão de sutura mais tradicional é a realização de suturas horizontais, que cruzam perpendicularmente o plano de lesão, com distância de 5 mm entre o ponto mais periférico e o central, e ancoragem meniscal a 5 mm da lesão. 26 Em 2012, Matsubara et al. 27 modificaram a técnica ao mostrar que as suturas cruzadas (ou em “X”) são mecanicamente superiores às suturas duplas paralelas, com maior tensão máxima e rigidez. Supõe-se que a orientação oblíqua dos pontos em relação às fibras colágenas circunferenciais proporciona a vantagem mecânica desse padrão de sutura.

Outra modificação técnica foi descrita por Bhatia et al. 28 ao associar uma ancoragem transóssea à sutura horizontal tradicional, na tentativa de melhorar a resistência mecânica do reparo, controlar a extrusão e ainda estimular biologicamente o reparo mediante fatores oriundos dos túneis ósseos. Nessa técnica, cada uma das bordas livres criadas pela lesão radial são laçadas em sua porção mais periférica, e essas duas suturas são trazidas por dois tuneis transósseos e feitas sobre um botão preso na cortical anterior da tíbia. O acréscimo da sutura transóssea à sutura das lesões radiais duplicou a resistência máxima à tração em comparação com a sutura isolada, e resultou em uma diástase entre os cotos meniscais significativamente menor. Essa técnica parece ser especialmente válida para o menisco medial, visto o seu caráter de menor mobilidade, mas ainda requer mais estudos acerca de eventuais efeitos de ancoragem no menisco lateral.

Recentemente, novos instrumentos de sutura *all-inside* foram lançados, e permitem uma sutura circunferencial do menisco. Essas suturas circunferenciais apresentaram menor abertura meniscal após teste cíclico com 100, 300 e 500 ciclos, maior rigidez e maior força até a falha. 20

## Tratamento das Lesões da Raiz Meniscal

As lesões de raiz são aquelas que ocorrem até 10 mm da inserção tibial meniscal, e apresentam grande relevância pelo seu comportamento biomecânico, que equivale ao de uma meniscectomia completa, diante da perda da tensão circunferencial meniscal e a consequente extrusão meniscal. Os efeitos associados, de aumento do pico de pressão e redução da área de contato, levam a uma história natural de progressão rápida do processo degenerativo e ao colapso do compartimento afetado. 29 30

As lesões podem ser divididas em traumáticas e degenerativas. As primeiras acometem mais frequentemente a raiz posterior do menisco lateral em pacientes jovens, e estão associadas a quadro de entorse aguda na lesão ligamentar, principalmente do ligamento cruzado anterior (LCA). As lesões degenerativas da raiz posterior do menisco medial podem ser classificadas em cinco padrões. 31

**Tipo 1–** Lesão estável parcial a um raio de 9 mm do centro da fixação da raiz;

**Tipo 2–** Lesão radial completa a um raio de 9 mm do centro da fixação da raiz;

**Tipo 3–** Alça de balde com deslocamento completo a um raio de 9 mm da fixação da raiz;

**Tipo 4–** Padrão complexo oblíquo a um raio de 9 mm da fixação da raiz; e

**Tipo 5–** Fratura por avulsão da raiz meniscal do platô tibial.

As lesões degenerativas são mais frequentemente encontradas em mulheres na quinta e sexta décadas de vida, e estão a associadas classicamente a estalido na região posterior do joelho após atividades de flexão excessiva do joelho ou descida de escadas. O joelho inicia um quadro de derrame articular moderado, que pode evoluir com dor importante a deambulação e dor na face interna do joelho, por lesão da raiz posterior do menisco medial. 31

**Fig. 1:**
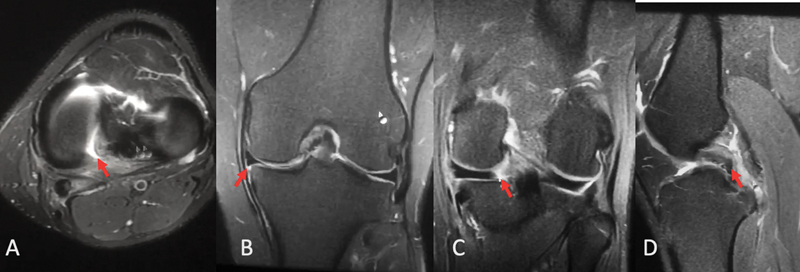
Ressonância magnética de paciente com lesão da raiz posterior do menisco medial: ( **A** ) aquisição axial; ( **B** ) aquisição coronal indicando extrusão; ( **C** ) lesão identificada na aquisição coronal; e ( **D** ) imagem na aquisição sagital.

O diagnóstico é realizado pelo histórico clínica associado a sinais específicos no exame de RM ( Fig. 1 ). O sinal do truncamento (perda de continuidade da porção posterior do menisco no corte coronal), o sinal do menisco fantasma (ausência da imagem meniscal no corte sagital), ou a imagem de lesão radial completa próxima à raiz são as imagens que identificam essa lesão. A extrusão meniscal, ou distância menor do que 3 mm entre a borda meniscocapsular e o limite do platô tibial, demonstra a perda da contenção circunferencial meniscal, e é sinal indireto da lesão da raiz. 32 Fazem parte da investigação diagnóstica a radiografia de rotina do joelho com carga e a radiografia panorâmica para a avaliação do eixo do membro inferior.

O princípio do tratamento é baseado no alívio sintomático, no restabelecimento da biomecânica articular e na prevenção da osteoartrite. O tratamento da lesão da raiz meniscal pode ser conservador ou cirúrgico. O tratamento conservador é reservado para pacientes idosos, sedentários, que já apresentam colapso do osso subcondral ou osteoartrite avançada. Em pacientes com extrusão meniscal avançada, má qualidade meniscal e obesos, a cirurgia é um procedimento de exceção. O tratamento não cirúrgico apresenta resultados insuficientes, com história natural de evolução progressiva do processo degenerativo, com taxas de evolução para artroplastia de 30 a 45% em 5 anos. 33

Os candidatos ideais para o tratamento cirúrgico são casos de lesões de origem traumática associadas a lesão ligamentar ou lesões degenerativas agudas, com pouca extrusão, em pacientes com lesões condrais Outerbridge 1 ou 2. Pacientes com desalinhamento do membro devem ter a cirurgia de alinhamento realizada no mesmo ato ou anteriormente ao tratamento da lesão meniscal.

Demonstrou-se que o tratamento cirúrgico recupera a biomecânica da distribuição de carga nativa do menisco após o reparo, melhora os escores funcionais e resulta em índices menores de conversão para artroplastia. 34 35

A sutura da lesão da raiz meniscal é feita por meio de técnica específica, que requer o uso de dispositivos especiais para transfixar os meniscos (pinças *all-inside* ou passadores de sutura tipo gancho), que passam fios ultrarresistentes pelo coto da raiz meniscal. Depois, utilizando guias específicos, túneis ósseos na região da raiz da tíbia são realizados, por onde os fios são transportados, com a fixação do coto meniscal no osso da tíbia, o que configura uma nova raiz meniscal. Esses fios são suturados na cortical anterior, e fixam os meniscos. O pós-operatório é altamente restrito, com limitação de carga e flexão excessiva do joelho por mais de seis semanas, seguida de carga progressiva e fortalecimento. 36

## Tratamento das Lesões Meniscais Degenerativas

A lesão meniscal degenerativa é uma condição caracterizada por mudanças progressivas na estrutura e na função dos meniscos do joelho, que resultam em deterioração ao longo do tempo. A degeneração meniscal é frequentemente associada ao processo natural de envelhecimento, que leva a uma diminuição na elasticidade e na resistência dos meniscos. Essa degeneração gradual torna os meniscos mais susceptíveis a lesões, até mesmo em atividades cotidianas de baixo impacto. Movimentos repetitivos, torções excessivas ou uma sobrecarga contínua podem resultar em rupturas ou outras lesões nos meniscos já degenerados. 37

O diagnóstico da lesão meniscal degenerativa é tipicamente realizado por meio de avaliação clínica e exames de imagem, como RM, que proporciona uma visualização detalhada das estruturas do joelho e auxilia na determinação da extensão da lesão. 38

O tratamento dessa condição é individualizado, conforme a gravidade dos sintomas, a idade do paciente, o nível de atividade física e o estado degenerativo global da articulação. A avaliação radiográfica é essencial para o entendimento da lesão como uma fonte isolada de dor ou como parte de um processo de degeneração articular mais difuso, além da avaliação do eixo do membro, pois a osteotomia pode ser uma estratégia importante no alívio da dor e no aumento da longevidade articular. 39 40

As estratégias de tratamento incluem repouso, crioterapia, terapia física para fortalecimento muscular, uso de medicamentos anti-inflamatórios não esteroides para o controle da dor e da inflamação. Na ausência de resposta, o tratamento articular com infiltrações com corticosteroides ou viscossuplementação são estratégias frequentemente utilizadas.

O tratamento cirúrgico das lesões degenerativas configura exceção, visto que diversos ensaios clínicos não mostraram resultados melhores após o tratamento cirúrgico. 9 10 39 40 41 Portanto, a cirurgia é mais bem indicada em casos de falência do tratamento conservador ou da presença de sintomas mecânicos articulares, como travamentos e bloqueios articulares, e deve ser realizada com a ressecção da menor massa meniscal possível para evitar complicações como progressão da artrose e osteonecrose pós-artroscopia. 37

A abordagem cirúrgica das lesões degenerativas com padrão horizontal envolve desafios especiais. O maior distanciamento entre as bordas da lesão e o maior grau de danos cartilaginosos associados afetam negativamente os resultados da cirurgia; além disso, a presença de sintomas mecânicos não é um fator preditivo de sucesso após a abordagem. 42 Quando indicada a meniscectomia, a ressecção completa do folheto inferior da lesão parece apresentar resultados clínicos inferiores em comparação com a ressecção parcial. 43 Também é possível executar técnicas de sutura circunferencial do menisco, que, embora possam estar associadas a taxas de cicatrização baixas, apresentam bons resultados clínicos. 44

## Tratamento das Lesões Meniscais em Rampa

As lesões meniscais em rampa acontecem na região periférica do corno posterior do menisco medial, e exigem um elevado índice de suspeição para o seu diagnóstico. Exames de imagem como a RM podem não apresentar sinais específicos de lesões que, com a exploração artroscópica, são diagnosticadas, de modo que uma rotina criteriosa de avaliação deve ser executada. 45 46 47

Esse tipo de lesão parece ter como fatores de risco o sexo masculino, a idade inferior a 30 anos, o edema tibial posteromedial na RM, lesões meniscais laterais concomitantes, lesões completas do LCA e lesões ligamentares crônicas. 48 Alguns estudos 49 50 51 sugerem que lesões meniscais em rampa estáveis dispensam a realização de sutura, e somente permitem abordagem com o debridamento associado a estabilização ligamentar. Nas lesões maiores do que 2 cm e com instabilidade detectada, o reparo por sutura meniscal deve ser realizado para evitar a permanência da instabilidade ou a falha da reconstrução. 47 49 51

A avaliação artroscópica se inicia com a análise da mobilidade do menisco medial com a palpação por meio de sonda; entretanto, o uso dos portais artroscópicos convencionais normalmente é insuficiente para o diagnóstico adequado das lesões, sendo necessária a adoção de visualização posterior pelo espaço entre o ligamento cruzado posterior e o côndilo femoral medial. Se necessário, deve ser realizado um portal artroscópico posterior para a palpação da transição menisco capsular, que pode ser utilizado para as técnicas de sutura indicadas. 47 52

As lesões em rampa podem ser classificadas em cinco tipos: 53

**Tipo 1–** Lesões meniscocapsulares. Essas lesões estão localizadas muito perifericamente. A mobilidade durante a palpação anterior com a sonda é baixa;

**Tipo 2–** Lesões parciais superiores. Essas lesões são estáveis e só podem ser diagnosticadas por visão posterior. Também apresentam baixa mobilidade durante o teste com a sonda;

**Tipo 3–** Lesões parciais inferiores ou ocultas. As lesões não são visíveis inicialmente com a visão posterior, mas podem ser fortemente suspeitadas quando há mobilidade significativa no teste com a sonda;

**Tipo 4–** Ruptura completa na zona vermelho-vermelha. A mobilidade na sondagem é muito alta;

**Tipo 5–** Lesão dupla.

**Fig. 2:**
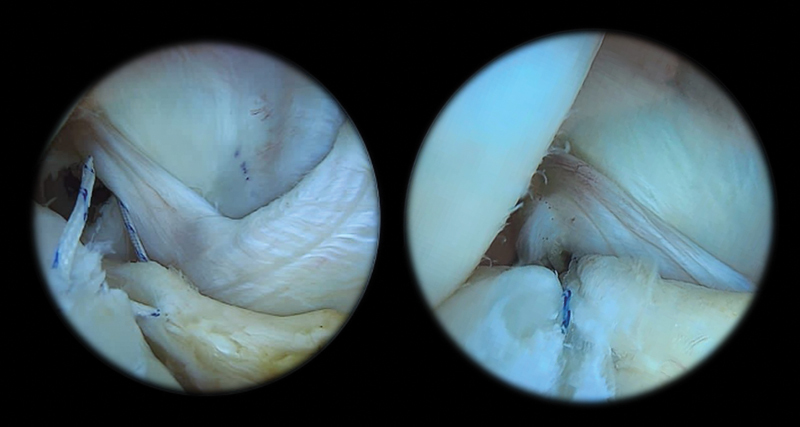
Visão através do intercôndilo de lesão meniscal em rampa com sutura da lesão pelo portal posterior.

Diferentes técnicas de reparo podem ser realizadas, incluindo a abordagem por um portal posteromedial com ganchos para sutura ( Fig. 2 ) ou pinças transfixantes. 47 54 A execução da sutura mediante dispositivos *all-inside* também pode ser uma opção de tratamento, embora exista uma preocupação com a possibilidade de falha devido ao não ancoramento dos dispositivos na cápsula e a possível necessidade de modificações técnicas para que se consiga promover adequadamente o reparo com esses dispositivos. 55 56 57 58

Embora nos últimos anos venha aumentando o número de estudos investigando o tratamento das lesões meniscais em rampa, há poucos estudos com nível de evidência elevado disponíveis, e mais estudos são necessários para esclarecer alguns questionamentos sobre o tema.

## Transplante Meniscal

O transplante meniscal é uma alternativa de salvamento que visa o alívio sintomático, a melhora funcional e o retardo do processo degenerativo articular, e é indicado em pacientes submetidos a meniscectomia total ou subtotal prévia. Entretanto, a escolha do paciente deve ser criteriosa.

Artrite inflamatória, imunodeficiência, infecção prévia e artrose grave são condições que contraindicam o procedimento; o aplainamento do côndilo ou a presença de osteófitos, embora não configurem contraindicação absoluta, estão associados a um prognóstico pior. Outras condições, como obesidade, instabilidade ligamentar, desvio de eixo e defeitos focais da cartilagem, devem ser corrigidas antes da cirurgia do transplante ou simultaneamente. 59 60

O histórico clínico deve ser complementado por exame físico com pesquisa de cicatrizes prévias, arco de movimento, instabilidade ligamentar, mau alinhamento e dor à palpação da linha articular, além de avaliação radiológica completa.

**Fig. 3:**
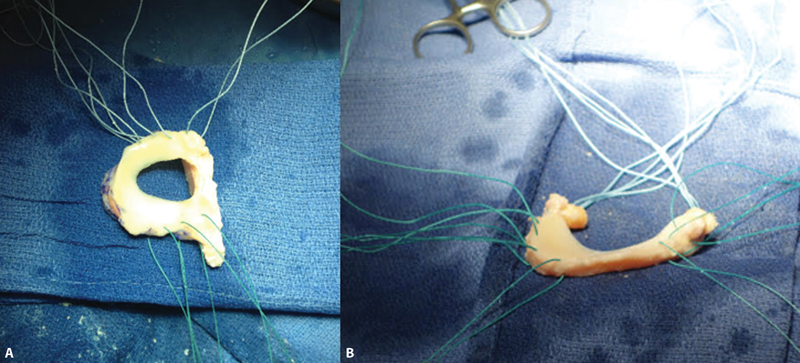
( **A** ) Aloenxerto do menisco lateral com crista óssea; ( **B** ) aloenxerto do menisco medial com *plugs* ósseos.

A técnica cirúrgica do transplante pode ser realizada por via aberta ou artroscópica, sendo que esta última apresenta menor morbidade. O tamanho do menisco é estimado utilizando-se radiografia nas incidências anteroposterior (AP) e de perfil. Os métodos de fixação mais utilizados são com *plug* ósseo sob as raízes meniscais, ou com crista óssea ( Fig. 3 ). Após a fixação óssea, o menisco deve ser suturado junto à cápsula articular mediante as diversas técnicas de sutura meniscais ( *all-inside* , *inside out* e *outside in* ). 61

## Reabilitação

O pós-operatório da meniscectomia artroscópica envolve a liberação de carga completa precoce, e pode-se adotar o uso de muletas por 1 a 2 semanas, de acordo com o grau de dor apresentado. O uso de joelheiras ou imobilizadores é dispensado, e a liberação precoce para ganho do arco de movimento é recomendada, e deve iniciar com um programa direcionado à recuperação dos déficits musculares e proprioceptivos, de modo a permitir o retorno ao treinamento e à prática esportiva. 62

O pós-operatório das suturas meniscais varia conforme o padrão da lesão e a técnica de sutura realizada. Nas lesões longitudinais, a carga axial pode ser liberada com o uso de imobilizadores do joelho ou joelheiras; entretanto, nas lesões radiais o paciente é orientado a não submeter o membro operado a cargas, para minimizar o risco da diástase meniscal ou ruptura da sutura, por 4 a 6 semanas. Nas primeiras 3 a 4 semanas, estimula-se o arco de movimento até 90° graus para evitar a rigidez articular, e, em seguida é liberado de modo completo, somente com a manutenção da limitação para realizar flexão profunda em cadeia cinética fechada por períodos de 4 a 6 meses. 62

## Considerações Finais

O tratamento das lesões meniscais vem evoluindo com o reconhecimento de padrões de lesões antes não diagnosticados, assim como com o desenvolvimento de materiais e técnicas que facilitam a realização das suturas meniscais. O tratamento conservador ainda parece uma boa estratégia nos pacientes com lesões degenerativas. A meniscectomia é evitada como medida inicial, salvo em situações nas quais as técnicas de sutura não seriam possíveis e quando há aumento do risco de degeneração articular em longo prazo. Lesões específicas representam um desafio à parte, que levarão ao desenvolvimento de novas estratégias de tratamento no futuro.
